# Asperuloside attenuates cadmium-induced toxicity by inhibiting oxidative stress, inflammation, fibrosis and apoptosis in rats

**DOI:** 10.1038/s41598-023-29504-0

**Published:** 2023-04-07

**Authors:** Zhiyang Kong, Chunhong Liu, Opeyemi Joshua Olatunji

**Affiliations:** 1 Second Peoples Hospital, Wuhu City, 241001 Anhui China; 2 Traditional Thai Medical Research and Innovation Center, Faculty of Traditional Thai Medicine, Prince of Songkla University, Hat Yai, 90110 Thailand; 3 African Genome Center, Mohammed VI Polytechnic University, Ben Guerir, 43150 Morocco

**Keywords:** Pharmacology, Toxicology

## Abstract

This present study investigated the protective effects of asperuloside (ASP) against cadmium-induced nephrocardiac toxicity. Rats were treated with 50 mg/kg of ASP for five weeks and CdCl_2_ (5 mg/kg, p.o., once daily) during the last 4 weeks of ASP treatment. The serum levels of blood urea nitrogen (BUN), creatinine (Scr), aspartate transaminase (AST), creatine kinase-MB (CK-MB), troponin T (TnT) and lactate dehydrogenase (LDH) were evealuted. Oxido-inflammatory parameters were detected via malondialdehyde (MDA), reduced glutathione (GSH), catalase (CAT), superoxide dismutase (SOD), tumor necrosis factor alpha (TNF-α), interleukin-6 (IL-6), interleukin-1beta (IL-1β) and nuclear factor kappa B (NF-κB)**.** Additionally, the cardiorenal levels of caspase 3, transforming growth factor-β (TGF-β), α-smooth muscle actin (α-SMA), collagen IV and Bcl2 were measured by ELISA or immunohistochemical assays. The results indicated that ASP significantly decreased Cd-instigated oxidative stress, serum BUN, Scr, AST, CK-MB, TnT and LDH as well as histopathological alterations. Furthermore, ASP notably attenuated Cd-induced cardiorenal and apoptosis and fibrosis by reducing caspase 3 and TGF-β levels, as well as reducing the stain intensity of a-SMA and collagen IV, while increasing Bcl2 intensity. These results revealed that ASP attenuated Cd induced cardiac and renal toxicity which may be attributed to reducing oxidative stress, inflammation, fibrosis and apoptosis.

## Introduction

Cadmium (Cd), a naturally occurring heavy toxic metal is found at low concentrations in the earth crust, however the concentration of this carcinogenic metal has increased tremendously in the atmosphere in recent years due to several factors including industrial (metal alloys, nickel-Cd batteries, polluted water, food and soil), agricultural (fertilizers) and environmental pollution (cigarette smoke). The stable divalent cation property of Cd makes it non-biodegradable, and possess significant health risk to human population^1,2^. Accumulating evidences have portrayed the multiple organ damages induced by Cd toxicity. Cd has been implicated in cardiac, renal, hepatic and testicular injuries^3,4^. In addition, chronic Cd exposure can lead to osteoporosis, bone fractures and anaemia^5^.

Oxidative stress accrued due to excessive generation of reactive oxygen species (ROS) is the chief culprit implicated in the pathogenesis of Cd induced muti-organ toxicity. Oxidative stress and ROS are capable of stimulating inflammatory cascade via generation of proinflammatory cytokines^6,7^. In addition, Cd can also disrupt sulfhydryl homeostasis culminating in suppression of antioxidant defense, while simultaneously promoting cellular apoptosis^8,9^. Mitigating the adverse toxicity of Cd is quite challenging due to the possible risk of toxicity progression involved in approaches like chelation therapy^9^. However, in recent years the antioxidant therapy has gain enormous attention in the prevention or treatment of drug/metal induced organ toxicity due to their inhibitory role on oxidative stress and inflammation^8,10,11^**.** Since the disruption in the oxidants and antioxidants balance as well as induction of inflammatory responses is a major mechanism involved in Cd-induced toxicity, the elimination of oxido-inflammation may be a viable therapeutic strategy to mitigate Cd toxicity.

Asperuloside (ASP) is an iridoid glycoside found in Rubiaceae plants and it has been reported to display intense anti-inflammatory, anticancer, antiobesity and antioxidant properties^12,13^. ASP alleviated oxido-inflammatory response in DSS-induced chronic colitis^14^. In another study ASP inhibited NF-κB and MAPK inflammatory pathways in acute lung injury^15^. In addition, Peng et al. reported that ASP displayed uroprotective effects against cyclophosphamide induced urotoxicity by modulating oxidative stress and inflammation^16^. However, no investigation is available on the potentials of ASP in mitigating Cd-induced nephro-cardiac toxicity. In this regard, this study investigated the nephro-cardiac protective role of ASP against Cd induced toxicity in rats.

## Materials and methods

### Chemicals and reagents

Asperuloside (purchased from Shanghai Yuanye Biotechnology Co., Ltd. Shanghai, China) was gratefully gifted by Professor Jian Tang, Bozhou University, China. Cadmium chloride (CdCl_2_)was obtained from Sigma-Aldrich Chemical Company, St. Louis, USA. All other chemicals used were of analytical grade.

### Experimental animals

This study was performed on 24 adult male Sprague Dawley rats (180–220 g). The rats were accommodated, fed and treated following the guidelines of the care and use of laboratory animals of the National Institutes of Health (NIH Publications No. 8023, revised 1978) and approved the Institutional Animal Ethics Committee of the Second Peoples Hospital of Wuhu City. The study is reported in accordance with ARRIVE guidelines.

### Experimental protocol

The rats were fed with a standard rodent chow (TROPHIC Animal Feed High-tech Co., Ltd. Nantong, China) and water ad libitum under standard animal husbandry conditions. Following seven days of acclimatization, the rats were divided into four groups of six rats each as follows: Normal control treated with normal saline for 5 weeks; ASP control group treated with 50 mg/kg ASP for 5 weeks; Cd control group treated saline for 5 weeks + 5 mg/kg CdCl_2_ during the last 4 weeks of treatment; ASP + Cd group treated with 50 mg/kg ASP for 5 weeks + 5 mg/kg CdCl_2_ during the last 4 weeks ASP treatment. CdCl_2_ and ASP were solubilized in saline and administered to the animals via oral gavage. The doses of ASP and CdCl_2_ used in this study were adopted from earlier studies^9,10^. After the last treatment, all the animals were fasted overnight and blood samples were obtained via cardiac puncture under thiopental anaesthesia. The blood was centrifuged to obtain the serum which was used for biochemical analysis. The animals were sacrificed by cervical dislocation, the heart and kidney tissues were excised, washed with cold normal saline and a large portion of the excised tissues was immediately frozen with liquid nitrogen and stored at − 80 °C for until further analysis, while the remaining portion of the harvested tissues were fixed in a 10% buffered formalin solution for histopathological and immunohistochemical studies.

### Estimation of serum nephrocardiac biomarkers

The serum concentrations of blood urea nitrogen (BUN), creatinine (Scr), aspartate transaminase (AST), creatine kinase-MB (CK-MB), troponin T (TnT) and lactate dehydrogenase (LDH) were determined using commercial assay kits from Jiancheng Bioengineering Institute (Nanjing, China) and Cusabio Technology (Wuhan, China) following the manufacturer’s protocols**.**

### Estimation of oxidative stress biomarkers

The renal and cardiac tissues were homogenised in 0.1 M phosphate buffer (10% w/v, pH 6.8), centrifuged (6000 g for 30 min at 4 °C) and the supernatant obtained from both tissues were subjected to the analysis of superoxide dismutase (SOD), catalase (CAT), reduced glutathione (GSH) and malonaldehyde (MDA) level were measured with biochemical assay kits (Jiancheng Bioengineering Institute, Nanjing, China) following the manufacturer’s protocol.

### Estimation of inflammatory and apoptosis markers

Likewise, the levels of tumor necrosis factor alpha (TNF-α), interleukin-6 (IL-6), interleukin-1beta (IL-1β) and nuclear factor kappa B (NF-κB) in the homogenates were assayed using standard ELISA method following the manufacturer’s protocol in the assay kit (Abcam, Cambridge, UK). Caspase 3 and TGF-β level were determined with ELISA kits from Cusabio Technology (China).

### Histopathological and immunohistochemical examination

The kidney and heart specimens stored in 10% buffered formalin were rinsed, dehydrated in alcohol, cleared using xylene and embedded in paraffin**.** The specimens were further sectioned and stained with eosin and haematoxylin (H&E). The stained slides were examined and photographed using a light microscope.

From the same specimens used for histological evaluation**,** the paraffin sections were deparaffinized and subjected to antigen retrieval. After blocking endogenous peroxidase with 3% H_2_O_2_, the sections were washed with PBS, incubated with primary antibodies (Ventana Medical Systems, Inc, USA) overnight at 4 °C followed by incubation with secondary antibodies for 2 h at room temperature. Thereafter, the tissue slices were stained with 3,3′-diaminobenzidine and counterstained with hematoxylin for 3 min. Brown granules represented immunohistochemical positive staining. The microscopic images of the tissues were quantitatively evaluated using the ImageJ software. The mean colour percentage area of the immunostained proteins were quantitatively assessed using ImageJ software (NIH, USA). Briefly ten non-overlapping fields per tissue section of each sample was randomly scanned to determine the relative area percentage of immunohistochemical expression levels of α-SMA, collagen IV and Bcl2 in different groups. The positive areas were calculated as a percentage of positively stained area divided by the total tissue area.

### Statistical analysis

Results were expressed as mean ± standard deviation and analyzed using GraphPad (version 9.0). Differences among the groups were analyzed using one-way ANOVA, followed by Newman–Keuls test for multiple comparisons. *P* < 0.05 was set as statistical significance.

### Ethics approval

The animal care and experiment procedures were conducted in accordance with the National Institutes of Health (NIH Publications No. 8023, revised 1978) guidelines and approved the Institutional Animal Ethics Committee of the Second Peoples Hospital of Wuhu City.

## Results

### Effects of ASP biochemical parameters

**Figure 1:**
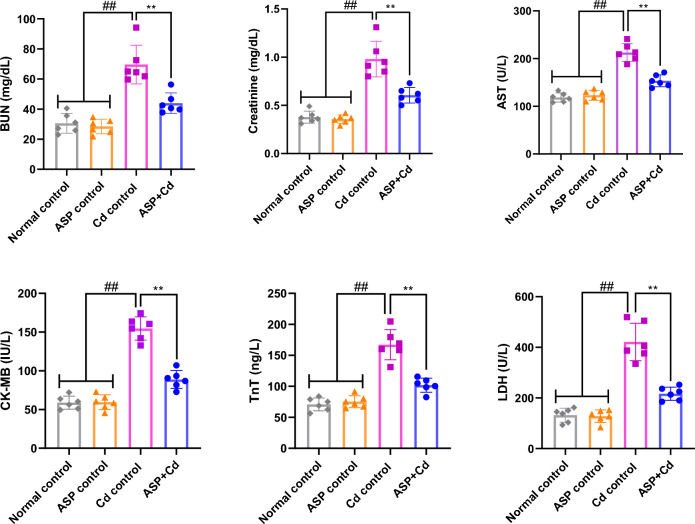
Effects of asperuloside on serum BUN, creatinine, AST, CK-MB, TnT and LDH of cadmium administered rats. Histograms represent the mean ± SD (n = 6). For all panels: ##*p* < 0.05 compared to normal and ASP control groups; **p < 0.05 compared to Cd control group.

The serum BUN and Scr of rats administered with Cd were significantly elevated when compared to the normal and ASP control groups (*p* < 0.05, Fig. 1). Interestingly, supplementation with ASP notably reduced the elevated levels of these kidney function parameters in comparison with the Cd group (*P* < 0.05). Meanwhile, the serum concentrations of AST, CK-MB, TnT and LDH were markedly higher in the Cd group, while treatment with ASP reversed the elevated levels of these biomarkers compared to the Cd group (Fig. 1).

### Effects of ASP of oxidative stress indices

**Figure 2:**
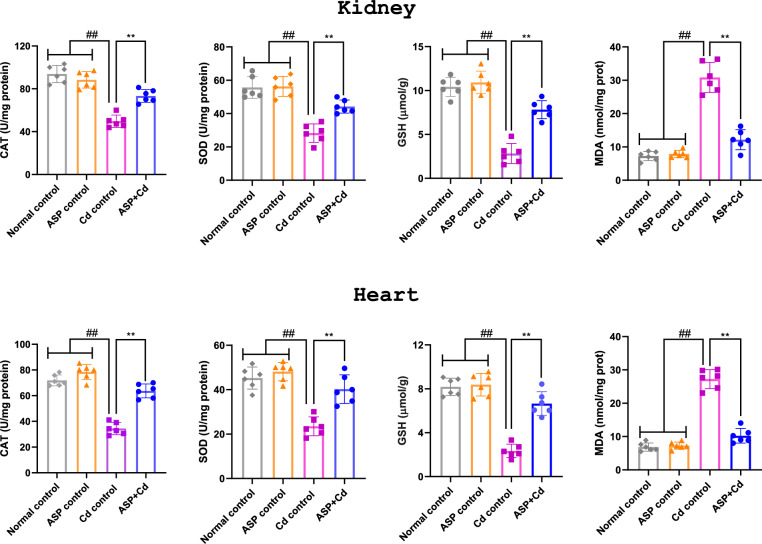
Effects of asperuloside on kidney and heart CAT, SOD, GSH and MDA levels of cadmium administered rats. Histograms represent the mean ± SD (n = 6). For all panels: ##*p* < 0.05 compared to normal and ASP control groups; **p < 0.05 compared to Cd control group**.**

The effect of ASP on hepatic and cardiac antioxidant enzymes levels are shown in Fig. 2. The Cd rats showed increased oxidative stress as observed by significantly reduced levels of CAT, SOD and GSH, while MDA level was remarkably elevated as compared with the normal and ASP control groups (Fig. 2, *p* < 0.05). However, in the animals treated with ASP, MDA level was significantly reduced, while the activities of SOD, CAT and GSH were notably increased compared to the Cd group (Fig. 2).

### Effects of ASP of inflammatory mediators

**Figure 3:**
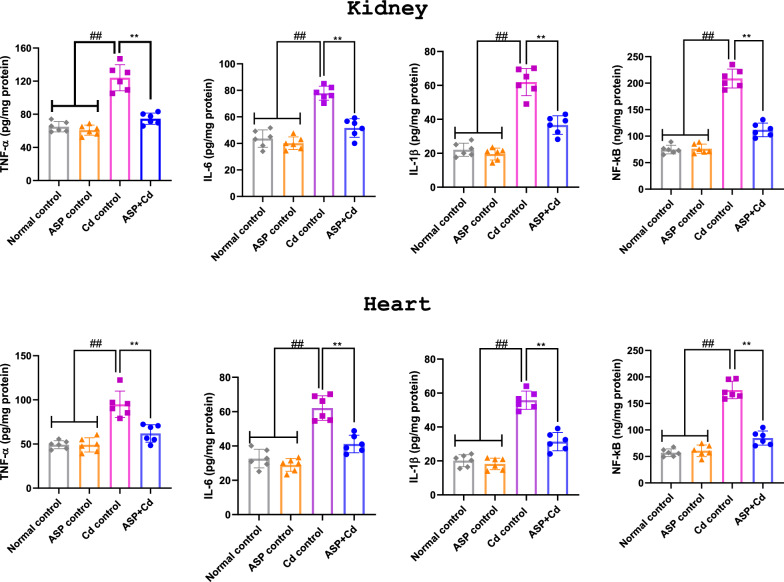
Effects of asperuloside on kidney and heart TNF-α, IL-6, IL-1β and NF-кB of cadmium administered rats. Histograms represent the mean ± SD (n = 6). For all panels: ##*p* < 0.05 compared to normal and ASP control groups; **p < 0.05 compared to Cd control group**.**

The exposure of rats to Cd for 4 weeks significantly increased nephro-cardiac levels of proinflammatory cytokines including TNF-α, IL-6 and IL-1β when compared to the control groups (Fig. 3). In contrast, the ASP treated rats showed significantly lowered concentrations of TNF-α, IL-6 and IL-1β when juxtaposed with the Cd group (Fig. 3). Furthermore, the results also showed that NF-кB level in the Cd group was markedly elevated (*p* < 0.05) when compared with that of normal and ASP control rats, while treatment with ASP significantly modulated NF-кB level in comparison to the Cd group (Fig. 3).

### Effects of ASP of caspase 3 and TGF-β

**Figure 4:**
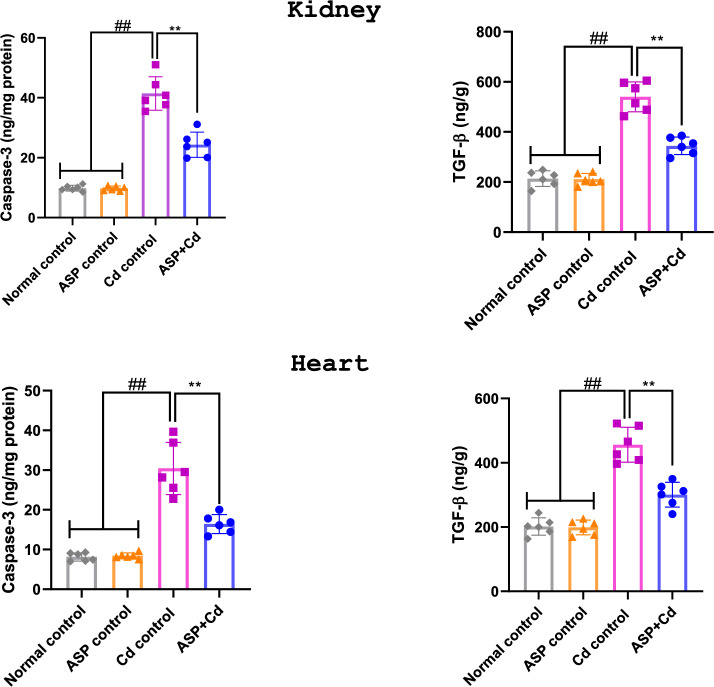
Effects of asperuloside on kidney and heart caspase 3 and TGF-β of cadmium administered rats. Histograms represent the mean ± SD (n = 6). For all panels: ##*p* < 0.05 compared to normal and ASP control groups; **p < 0.05 compared to Cd control group**.**

The administration of Cd for four weeks significantly (*p* < 0.01) increased cardiorenal levels of caspase 3 and TGF-β in the Cd group when compared to the control groups (Fig. 4). Whereas, treatment with ASP (50 mg/kg) significantly reduced Cd-mediated increase in caspase 3 and TGF-β when compared to the Cd group (Fig. 4).

### Effects of ASP on histopathological and immunohistochemical analysis

**Figure 5:**
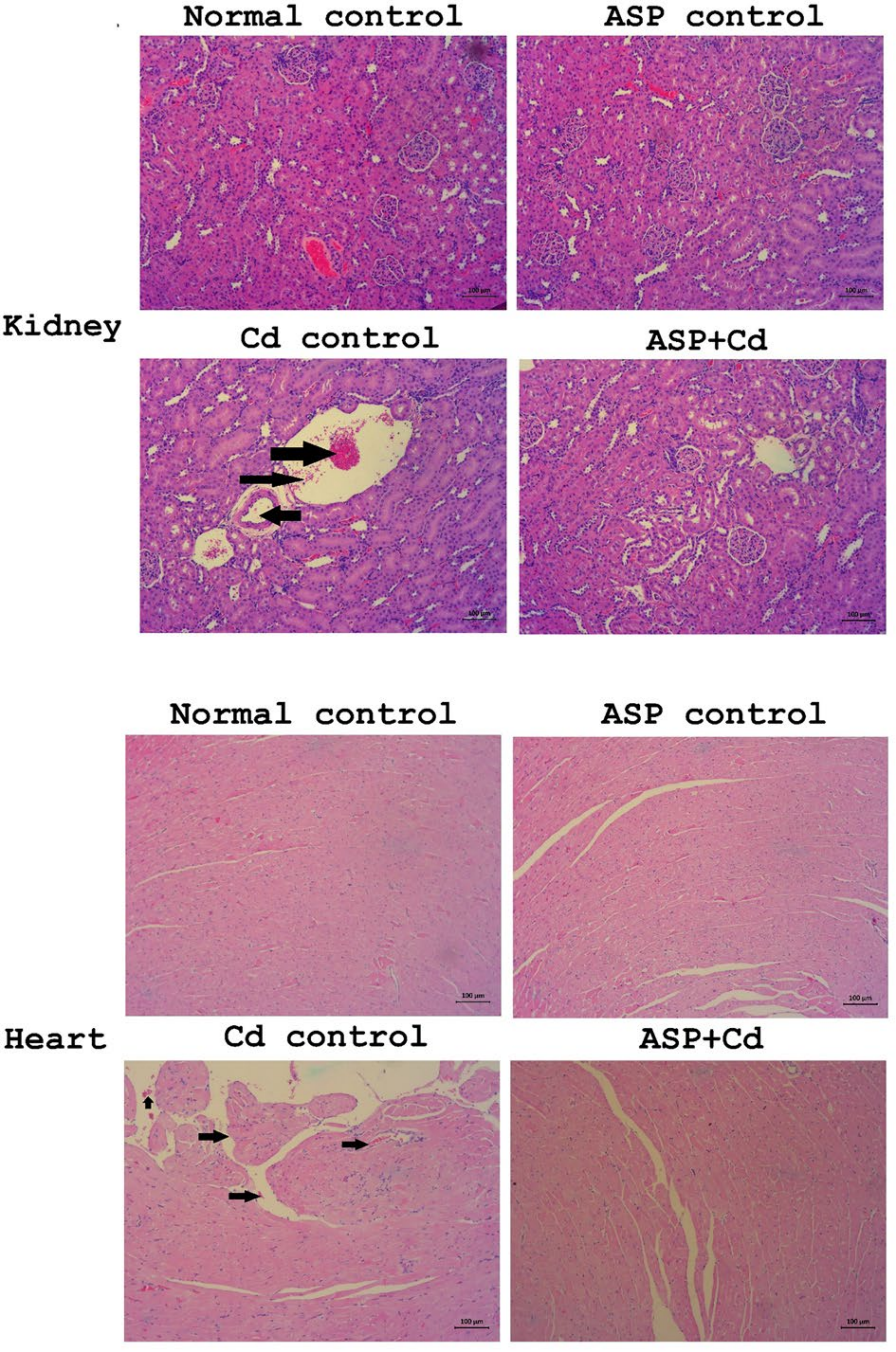
Histopathological changes in the kidney and heart tissues of cadmium administered rats**.** Black arrows: renal tubule and aggregates of inflammatory cells. X 400. Bar = 100 µm.

H&E stained renal tissues of the Cd group showed gross alterations, including glomerular shrinkage and atrophy, mesangial expansion and inflammatory cells infiltration as well as significant renal tubule disarrangement when compared with the normal and ASP control groups (Fig. 5). In contrast to the Cd rats, the renal histopathological alterations were markedly reduced in the ASP treated groups (Fig. 5).

Furthermore, the cardiac tissues of the Cd group showed disordered cardiac myofibers, congestion of blood vessels in the myocardium with significant inflammatory cells infiltration and necrotic myocyte when compared to the normal groups with distinctly arranged and structured myocardial fibers and cardiomyocytes (Fig. 5). On the other hand, the H&E section of the heart tissues from the ASP treated rats showed significant attenuation of the histopathological damages observed in Cd alone administered group (Fig. 5).

**Figure 6:**
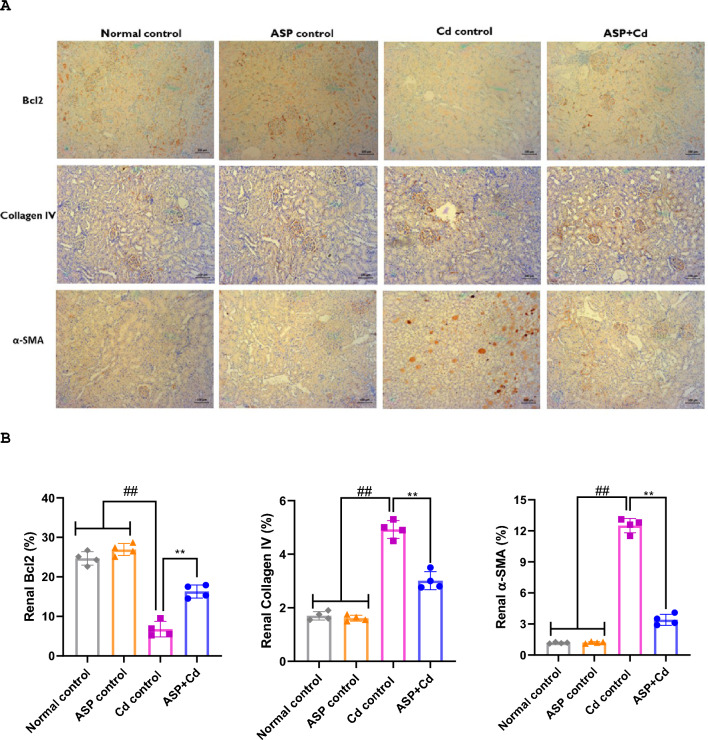

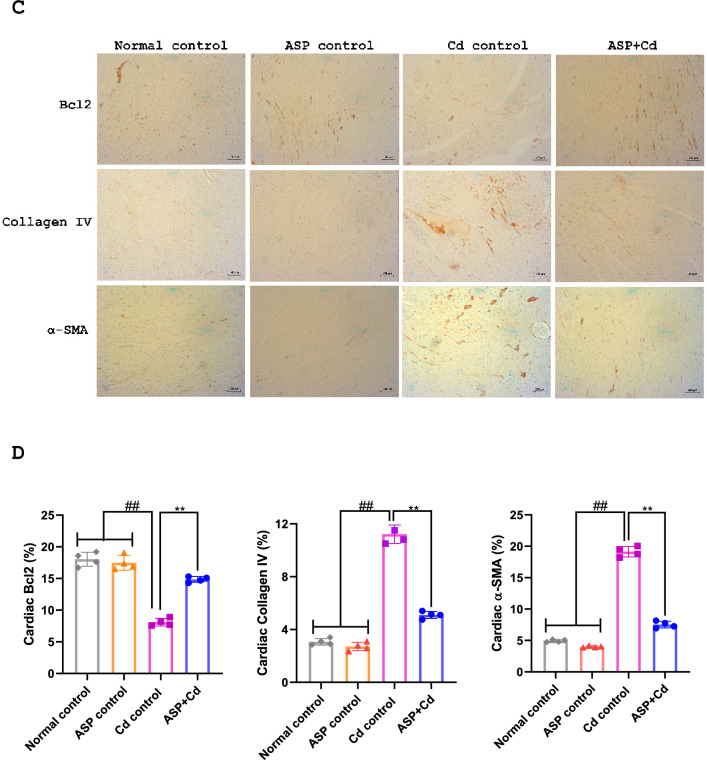
Immunohistochemical staining of (**A**) Bcl2, collagen IV and α-SMA, (**B**) quantitative levels of Bcl2, collagen IV and α-SMA in the kidney tissues of cadmium administered rats. Immunohistochemical staining of (**C**) Bcl2, collagen IV and α-SMA, (**D**) quantitative levels of Bcl2, collagen IV and α-SMA in the heart tissues of cadmium administered rats Data were presented as the mean ± SD (n = 4). ##*p* < 0.05 compared to normal and ASP control groups; **p < 0.05 compared to Cd control group.

The immunohistochemical protein expression of Bcl2, α-SMA and collagen IV in the renal and cardiac tissues are displayed in Fig. 6. When compared to the normal and ASP control groups, there were significant increases in the stain intensities of α-SMA and collagen IV, while the stain intensity of Bcl2 was markedly reduced in the Cd group. Whereas, ASP treatment notably increased the Bcl2 stain intensity and subsequent decrease in the α-SMA and collagen IV accumulation when compared to the Cd group (Fig. 6).

## Discussion

Renal and cardiac toxicities are two of the most frequently encountered negative health impact associated with cadmium exposure**.** Cd is a toxic heavy metal that is widely spread in the environment owing to numerous human activities, including industrial and agricultural pollutions. The existence of this metal in the environment is very harmful to living organisms such as plants, animals and human beings^17,18^**.** Cd can be found in the soil, vegetables, fruits as well as in contaminated water, and the inhalation/ingestion of these contaminated food or water samples results in various organ toxicities, especially when consumed over a long period of time^17,19^. Multiple studies have shown that the heart, liver, kidney and testes are major target organs of Cd toxicity^20–22^**.** Hence, this study investigated the role of ASP in ameliorating Cd induced nephro-cardiac toxicity in rats**.**

The findings from this study indicated that Cd-intoxication led to significant increases in the serum levels of BUN and creatinine. These kidney biochemical parameters are considered as standard biomarkers implicated in renal damage. In the event of kidney damage, these markers are leaked into the blood stream leading to their elevated levels^17,23^. Treatment with ASP significantly decreased the levels of these renal damage indicators. Meanwhile, significant increases were observed in the serum levels of TnT, LDH, CM-KB and AST after Cd intoxication. Generally, TnT, LDH, CM-KB and AST are domiciled in the cardiomyocytes cytoplasm under normal physiological conditions. However, in the evident of cardiac damage or insult, these enzymes are also released into the blood stream leading to their elevated levels in the serum. As such, these biomarkers are considered as an indicator of myocardial damage^24,25^. The co-administration of ASP with Cd notably reduced the levels of these cardiac biomarkers. These results were further validated by histopathological analysis of the nephro-cardiac tissues of the animals exposed to Cd toxicity which revealed severe pathological defects including necrosis, infiltration of inflammatory cells and disordered structural architecture, while treatment of Cd intoxicated animals with ASP reduced these alterations to near normal.

The main pathophysiological mechanism mediating Cd toxicity has been hinged on reactive oxygen species and oxidative stress mediated damages. It is well known that prolonged exposure to toxicants like heavy metals such as Cd strikes an avalanche of ROS production in the mitochondria which triggers series of pathways linked to oxidative stress^26–28^. Cd indirectly induces the formation of ROS including superoxide, hydrogen peroxide, nitric oxide and hydroxyl radicals, leading to electron transport chain inhibition in the mitochondria, depletion of intracellular GSH and lipid peroxide generation^4^. Much of the reported toxicity mediated by Cd is due to redox imbalance especially decreased GSH levels, antioxidant enzymes activities and increased lipid peroxidation. In fact, lipid peroxidation is thought to be a major hallmark of Cd-induced oxidative stress, which has been shown to be directly proportional to the extent or period of exposure^4,29^. In a recent study, Cd toxicity magnified protein oxidation and lipid peroxidation, while concurrently reducing antioxidant enzymes activities (SOD, CAT and GPx) in rats kidney^30^. Elmallah et al. also reported increased levels of MDA, with corresponding suppression of antioxidant enzymes in the testes of rats exposed to Cd toxicity^31^. In consonance with these reports, Cd intoxication led to drastic reduction in GSH level and the activities of SOD and CAT, while MDA was markedly increased. Interestingly, ASP played potent antioxidant effects by reducing lipid peroxidation (MDA) and increasing the activities of SOD, CAT, as well as GSH level in the treated rats.

The cordial relationship that exist between inflammation and oxidative stress has been a driving force in the pathophysiological outcomes mediated by these two events. Often times ROS induced oxidative stress has been shown to initiate series of pathways liked to inflammation. These inflammatory responses can further accrue additional ROS generation, leading to a viscous oxido-inflammatory cycle^32,33^. Specifically, Cd toxicity has been widely reported to be associated with inflammatory responses in immune cells through the activation of nuclear factor kappa B (NF-κB), which subsequently results in the elevated levels proinflammatory cytokines particularly TNF-α, IL-6 and IL-1β, culminating in acute/chronic inflammation and tissue damage^34,35^. In this study, the levels of proinflammatory cytokines including TNF-α, IL-1β and IL-6, as well as NF-κB were significantly elevated levels in the cardiac and renal tissues after exposure to cd toxicity. Whereas, ASP abrogated Cd-induced inflammatory response and prevented cardiorenal damage by reducing the levels of these inflammatory mediators.

Cd has also been shown to enhance ROS/oxidative stress mediated apoptosis, leading to tissue damages. Specifically, Cd targets the mitochondria through the activation of several intrinsic and extrinsic apoptotic pathways including caspases and proapoptotic proteins. Earlier studies have indicated that p53, caspase 3 and Bax are upregulated in response to Cd toxicity, while antiapoptotic Bcl-2 protein is downregulated^36,37^. In this study, it was observed that Cd-induced toxicity increased the level of caspase 3, while the expression of Bcl-2 protein was significantly reduced. ASP notably reversed these changes in the treated rats.

In this study, the level of TGF-β1 was significantly increased as well as the immuno-stained expression of α-SMA and collagen IV were dramatically increased in Cd-exposed rats, whereas coadministration with ASP remarkably ameliorated these markers of cardiorenal fibrosis. Accumulating evidences have shown that fibrosis and the deposition of extracellular matrix (ECM) plays a vital role in Cd induced cardiorenal toxicity^27,38^. TGF-β pathway is critically involved in interstitial fibrosis progression and it accelerates the onset and progression of glomerulosclerosis, tubulointerstitial and cardiomyocytes fibrosis via differentiation of myofibroblast and ECM deposit, resulting in accelerated collagen and α-SMA accumulation^27,38^. In addition, TGF-β1 can also induce increased production of ROS and inflammation^39^. Interestingly, treatment with ASP inhibited cardiorenal fibrosis in rats through the suppression of TGF-β level as well as α-SMA and collagen IV expression.

## Conclusions

In conclusion, this study portrayed that Cd toxicity enhanced in the production of oxidative stress, apoptosis and inflammation in the cardio-renal tissues of the rats. In addition, Cd also induced cardiorenal fibrosis by enhancing TGF-β/α-SMA/collagen IV expression. Whereas, ASP treatment alleviated Cd-triggered cardiac/nephrotoxicity by modulating oxidative stress, inflammation, apoptosis and fibrosis. Therefore, ASP may be considered as a potential agent to prevention of Cd-mediated toxicity.

## Data Availability

The data are available from the corresponding author on reasonable request.
