# Author Correction: Asperuloside attenuates cadmium-induced toxicity by inhibiting oxidative stress, inflammation, fibrosis and apoptosis in rats

**DOI:** 10.1038/s41598-024-51821-1

**Published:** 2024-02-13

**Authors:** Zhiyang Kong, Chunhong Liu, Opeyemi Joshua Olatunji

**Affiliations:** 1 Second Peoples Hospital, Wuhu City, 241001 Anhui China; 2 Traditional Thai Medical Research and Innovation Center, Faculty of Traditional Thai Medicine, Prince of Songkla University, Hat Yai, 90110 Thailand; 3 African Genome Center, Mohammed VI Polytechnic University, 43150 Ben Guerir, Morocco

Correction to: *Scientific Reports* 10.1038/s41598-023-29504-0, published online 07 April 2023

The original version of this Article contained errors in Figure 6A. During the assembly of the figure, one of the representative images was inadvertently duplicated for two different groups. For the collagen IV staining, the image for the ASP group was erroneously repeated for the ASP + Cd group. For the alpha-SMA staining, the image for the normal group was mistakenly repeated for the ASP group. The original Figure 6 and the accompanying legend appear below.

**Figure 6:**
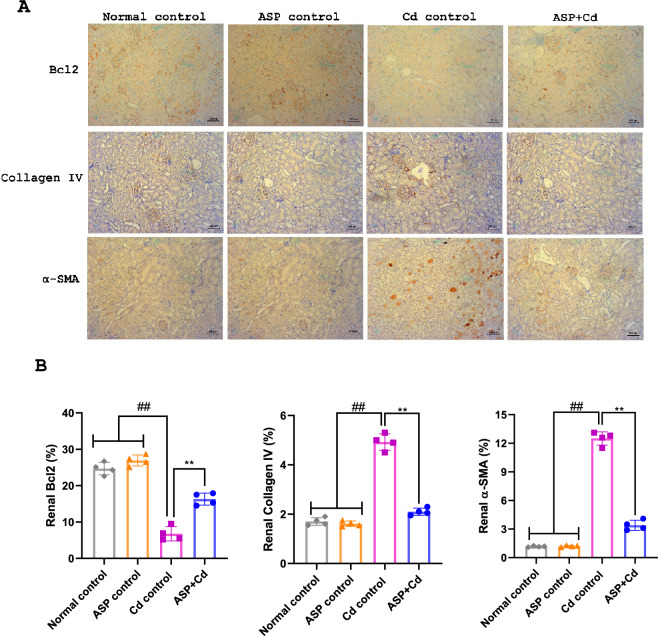

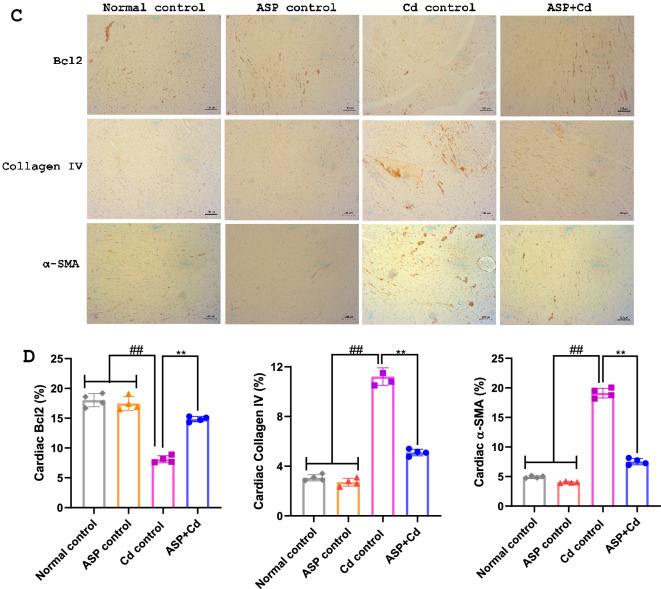
Immunohistochemical staining of (**A**) Bcl2, collagen IV and α-SMA, (**B**) quantitative levels of Bcl2, collagen IV and α-SMA in the kidney tissues of cadmium administered rats. Immunohistochemical staining of (**C**) Bcl2, collagen IV and α-SMA, (**D**) quantitative levels of Bcl2, collagen IV and α-SMA in the heart tissues of cadmium administered rats Data were presented as the mean ± SD (n = 4). ^##^*p* < 0.05 compared to normal and ASP control groups; **p < 0.05 compared to Cd control group.

The original Article has been corrected.

